# 3D Hydrogen Titanate Nanotubes on Ti Foil: A Carrier for Enzymatic Glucose Biosensor

**DOI:** 10.3390/s20041024

**Published:** 2020-02-14

**Authors:** Lulu Ma, Zhao Yue, Guona Huo, Shasha Zhang, Baolin Zhu, Shoumin Zhang, Weiping Huang

**Affiliations:** 1 The Key Laboratory of Advanced Energy Materials Chemistry (MOE), and TKL of Metal and Molecule-based Material Chemistry, College of Chemistry, Nankai University, Tianjin 300071, China; 15510956697@163.com (L.M.); 15230153709@163.com (G.H.); 2120170933@mail.nankai.edu.cn (S.Z.); zhangsm@nankai.edu.cn (S.Z.); 2 Hebei Normal University of Science & Technology, Hebei 066004, China; 3 Department of Microelectronics, Nankai University, Tianjin 300350, China; yuezhao@nankai.edu.cn; 4 College of Chemistry, National Demonstration Center for Experimental Chemistry Education (Nankai University), Tianjin 300071, China

**Keywords:** Ti foil, hydrogen titanate nanotubes, 3D structure, glucose oxidase, glucose biosensor

## Abstract

Glucose oxidase (GOx) based biosensors are commercialized and marketed for the high selectivity of GOx. Incorporation nanomaterials with GOx can increase the sensitivity performance. In this work, an enzyme glucose biosensor based on nanotubes was fabricated. By using Ti foil as a carrier, hydrogen titanate nanotubes (HTNTs), which present fine 3D structure with vast pores, were fabricated in-situ by the hydrothermal treatment. The multilayer nanotubes are open-ended with a diameter of 10 nm. Then glucose oxidase (GOx) was loaded on the nanotubes by cross-linking to form an electrode of the amperometric glucose biosensor (GOx/HTNTs/Ti electrode). The fabricated GOx/HTNTs/Ti electrode had a linear response to 1–10 mM glucose, and the response time was 1.5 s. The sensitivity of the biosensor was 1.541 μA·mM^−1^·cm^−2^, and the detection limit (S/N = 3) was 59 μM. Obtained results indicate that the in-situ fabrication and unique 3D structure of GOx/HTNTs/Ti electrode are beneficial for its sensitivity.

## 1. Introduction

Diabetes is widely recognized as one of the leading causes of death and disability [1]. Closely monitoring the blood glucose level of patients is necessary for preventing and reducing diabetes. Minimally invasive interventional blood glucose detection is the most promising and potential method. It is mainly based on electrochemical methods. For an electrochemical glucose sensor, the bioreceptor is the core part and the performance is closely related to it.

It is well known that enzymes are highly efficient and specific biocatalytic molecules [2]. Enzyme biosensors, using an enzyme as the molecular recognition element, have the advantages of high selectivity, high sensitivity and a fast response. The enzyme immobilization technique involves binding the target enzymes at a certain place through physical or chemical ways [3]. The immobilization technique limits the free flow of enzyme molecules. Immobilized enzymes have the following advantages: (i) improved thermal and operational stability compared with free enzymes, (ii) improved convenience of separating reaction products from mixtures and (iii) easy recovery by removing the enzyme from the reaction mixture, thus reducing the cost [4]. The development of materials science has led to syntheses of various materials for enzyme immobilization. Many researchers have studied the immobilization of enzymes onto carriers [5], a framework [6] or nanoparticles [7], in order to limit the movement of enzyme and protect it from the impact of the corrosive environment, so as to delay the denaturation of the enzyme. The research reports generally indicated that the choice of support materials was very important. The support materials must have strong consistency, good durability, convenient production and low price.

Nanomaterials are a popular carrier because of their large surface area [8,9]. At present, the application of enzyme electrodes based on nanomaterials has been widely explored [10]. The most common nanomaterials are noble metal nanoparticles, carbon materials (carbon nanotubes, graphene, carbon felt, etc.) and metal oxide nanomaterials [11,12,13].The application of noble metal nanomaterials is limited by their high price [14]. The application of the carbon nanotubes is limited by their highly toxic to cells of living body. Moreover, the hydrophobic nature of the carbon nanotubes makes it difficult to immobilize GOx properly [15]. Liu fabricated a graphdiyne (GDY) based composite with dual-enzyme activity by immobilizing ferrous ion and glucose oxidase onto GDY sheet [16]. However, the linear range is relatively narrow.

Nanostructured TiO_2_ has important applications in optoelectronic devices, sensors and energy conversion devices [17,18,19]. It has also been reported that TiO_2_ nanocomposites provide a new way to immobilize glucose oxidase and prepare biosensors [20]. Especially, tubular TiO_2_ with large surface area can provide more active sites for the immobilization of enzymes. It also can improve the electron transfer rate between the enzyme’s active center and the electrode, realizing the direct electron transfer [21]. The fouling substances produced in the process of bioelectrochemical detection can be photocatalytically decomposed into CO_2_ and H_2_O on TiO_2_ by ultraviolet or visible light irradiation to achieve self-cleaning [22,23,24]. All these make the nanostructured TiO_2_ an ideal enzyme immobilization carrier material for the enzyme electrodes. In our previous research work, TiO_2_ powder can be easily changed to a tubular structure by the hydrothermal method. The fabricated nanotubes were of high quality, with a diameter of about 10 nm [25,26,27].

Titanium foil was chosen as a suitable substrate for the TiO_2_ nanomaterial carrier because it could efficiently transmit electrons to the external circuit current. Moreover, titanium metal is a biocompatible metal [28]. It has good compatibility to human tissue and body fluids (non-toxic, non-allergenic and abnormal metabolism, no irritation to tissue). In addition, Ti has corrosion resistance and chemical stability, which enable it to be fabricated as implantable biosensors. In this paper, hydrogen titanate nanotubes (HTNTs) were in-situ fabricated on the surface of the Ti foil by a simple hydrothermal process. The GOx was immobilized to the HTNTs/Ti electrode by the cross linking method. Then the electrode based on the modified Ti foil was used as a transducer, and an electrochemical workstation was used as detector to evaluate the electrochemical performances for glucose.

## 2. Experiments

### 2.1. Reagents

The reagents (include butyl titanate, ethanol, concentrated sulfuric acid, concentrated hydrochloric acid, PBS and glucose) were of analytic grade and used without any further purification. The purity of titanium foil (0.127 mm thick, Alfa Aesar) was 99%. The purity of BSA was 96%. And GOx (10,000 GODU/g) was used. Deionized water was used throughout the experiments.

### 2.2. Preparation of the Glucose Biosensor Based on the HTNTs

HTNTs were directly synthesized on Ti foils by a hydrothermal treatment. The polished Ti foils (8 mm × 8 mm square) were firstly treated with concentrated sulfuric acid (H_2_SO_4_) and concentrated hydrochloric acid (HCl) mixed solution, and then soaked in titanium solution [24]. After calcination at 400 °C for 2 h, the obtained foils were hydrothermally treated in 10 M NaOH solution at 150 °C for 10 h. Then the samples were soaked in water, dilute nitric acid and water successively until the solution became neutral. The treated foils were dried in the air to obtain an HTNTs/Ti electrode.

An optimized cross-linking technique [29] was carried out to immobilize the GOx onto the HTNTs/Ti electrode. 25 mg BSA and 100 μL glutaraldehyde were dissolved in 1 mL PBS and mixed evenly. Then 100 uL mixed solution was deposited onto the surface of the HTNTs/Ti and dried at room temperature. 12.5 mg GOx was dissolved in 500 μL of PBS and mixed evenly. Then 100 uL of the mixed solution was deposited on the HTNTs/Ti electrode surface, and dried in the refrigerator at 4 °C overnight to obtain the GOx/HTNTs/Ti electrode.

### 2.3. Characterization Techniques

X-ray powder diffraction (XRD) pattern was obtained by using a RigakuD/MAX-2500 X-ray diffractometer with a working voltage and current at 40 kV and 40 mA, respectively. Morphologies of samples were characterized by scanning electron microscope (SEM, JEOL JSM-7500F) at 10.00 kV and transmission electron microscopy (TEM, FEI T20) at 200 kV. The chemical composition and oxidation state of elements on the surface of samples were identified by X-ray photoelectron spectroscopy (XPS; Kratos Axis Ultra DLD), and the binding energy was calibrated by taking C 1s peak at 284.6 eV as reference. Electrochemical experiments were performed with an electrochemical workstation (Zahner Zennium, Germany). A three-electrode system was used in this process, which employed the GOx/HTNTs/Ti electrode as the working electrode, a platinum sheet as the counter electrode, and an Ag/AgCl electrode as the reference. Cyclic voltammetric (CV) and amperometric response (I-t) were used to study the electrochemical performance of the electrode.

## 3. Results and Discussion

### 3.1. Surface Morphology

**Figure 1:**
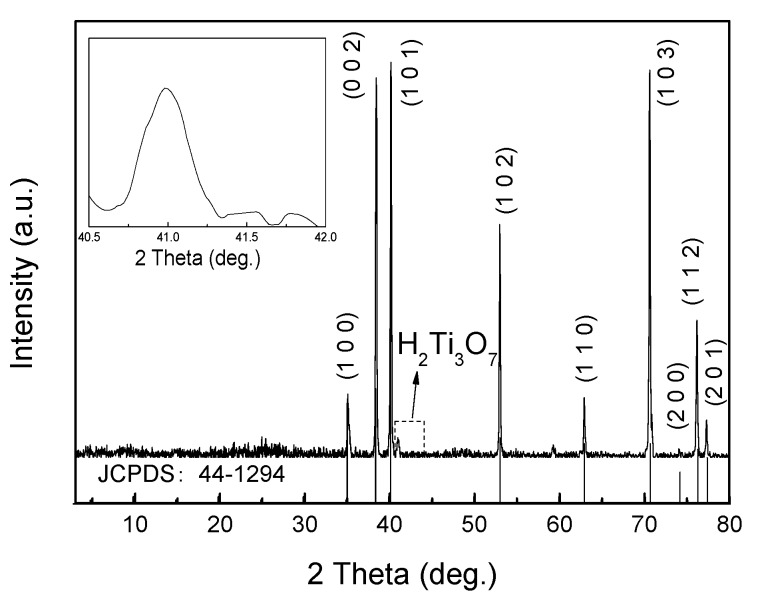
XRD pattern of the hydrogen titanate nanotubes (HTNTs)/Ti (inset: the curve is the enlarged XRD pattern).

Figure 1 shows the XRD pattern of the fabricated HTNTs/Ti. The superior peaks were observed mainly as diffraction planes of Ti (JCPDS No. 44-1294). As the samples were synthesized on top of Ti foils, the titanium peaks were very high and sharp. Apparently, titanate hydrate could be observed. As shown in the inserted graph of Figure 1, there was a visible diffractions peak located at 2*θ* = 41.0°, which was attributed to the (6 0 -3) diffraction of H_2_Ti_3_O_7_ (JCPDS No. 47-0561). It seemed that HTNTs was successfully fabricated on the Ti foil after the hydrothermal treatment.

**Figure 2:**
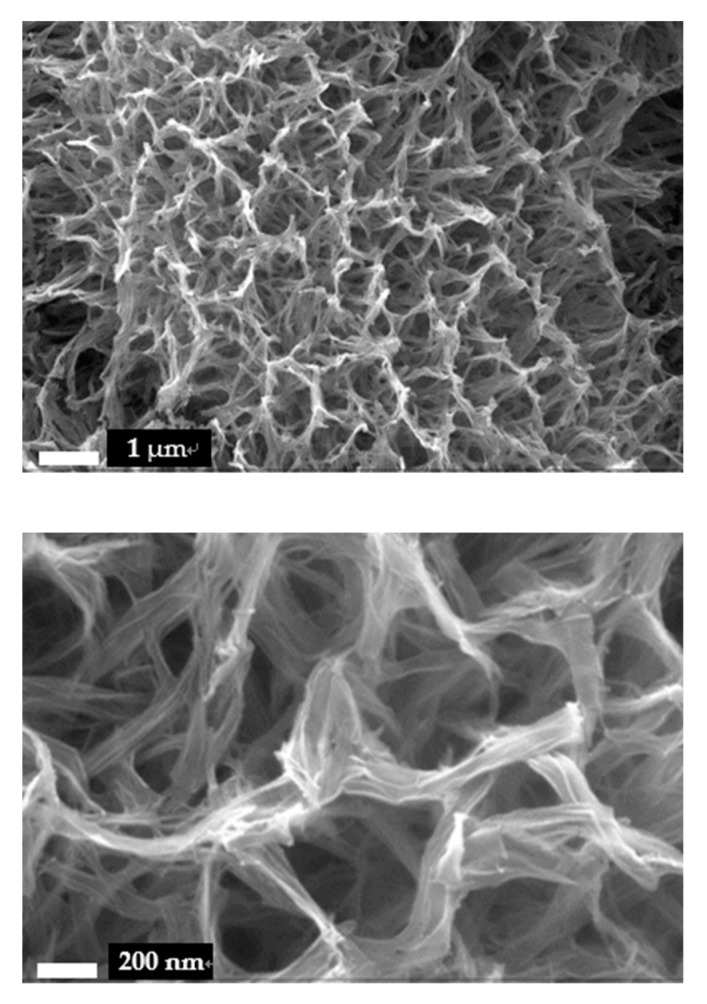
SEM images of the top view of HTNTs/Ti.

The surface morphology of Ti foil after the hydrothermal treatment was investigated by SEM, as shown in Figure 2. It can be observed that the surface of Ti foil was fully covered by 1D nanomaterials. The 1D materials intertwined together, and formed fine 3D structure with vast pores. The length of the 1D nanomaterials was more than hundreds of nanometers. Compared with the array structure made by the anodic oxidation method [30], HTNTs/Ti provided a larger surface area and stronger adsorption capacity. That could provide more effective immobilization for GOx.

**Figure 3:**
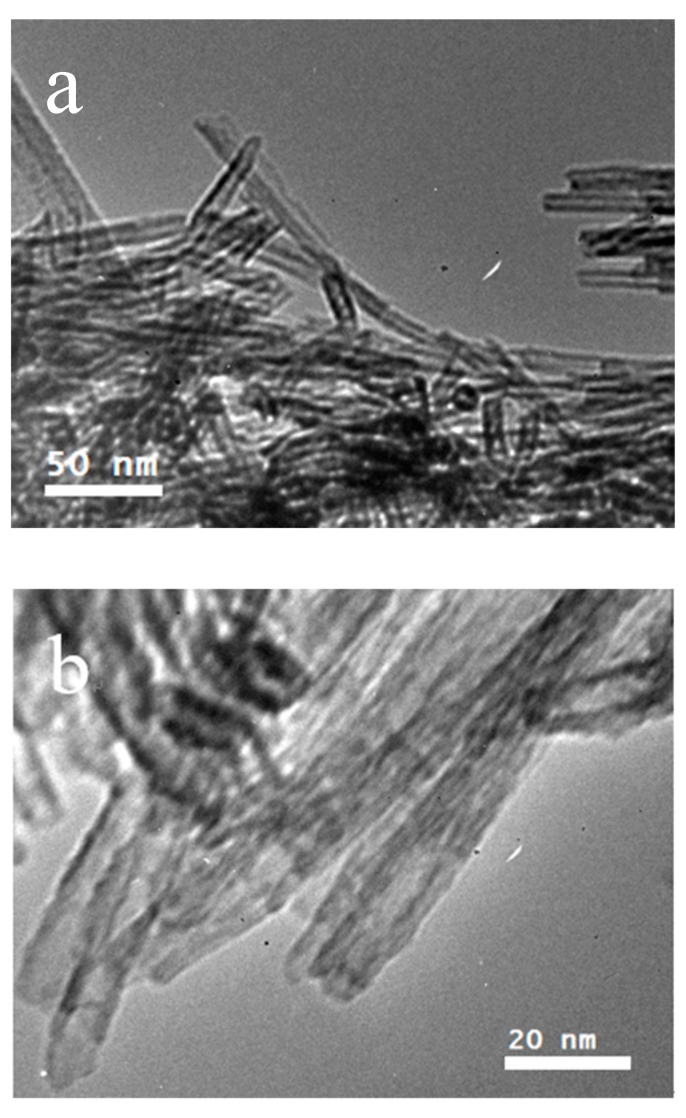
TEM (**a**) and HR-TEM (**b**) images of HTNTs on Ti foils.

The detailed structure of the 1D materials fabricated on the Ti foils was further observed by TEM. For this, some of the HTNTs were scraped off from the surface of the Ti foil and then dispersed in alcohol. Figure 3 shows the TEM images of the 1D nanomaterials. It can be clearly seen that the sample shows a 1D and tubular structure. The multilayer nanotubes were open-ended with an outer diameter of about 10 nm and an inner diameter of about 5 nm. So, the structure of the Ti foil surface was a network made of HTNTs of high quality.

**Figure 4:**
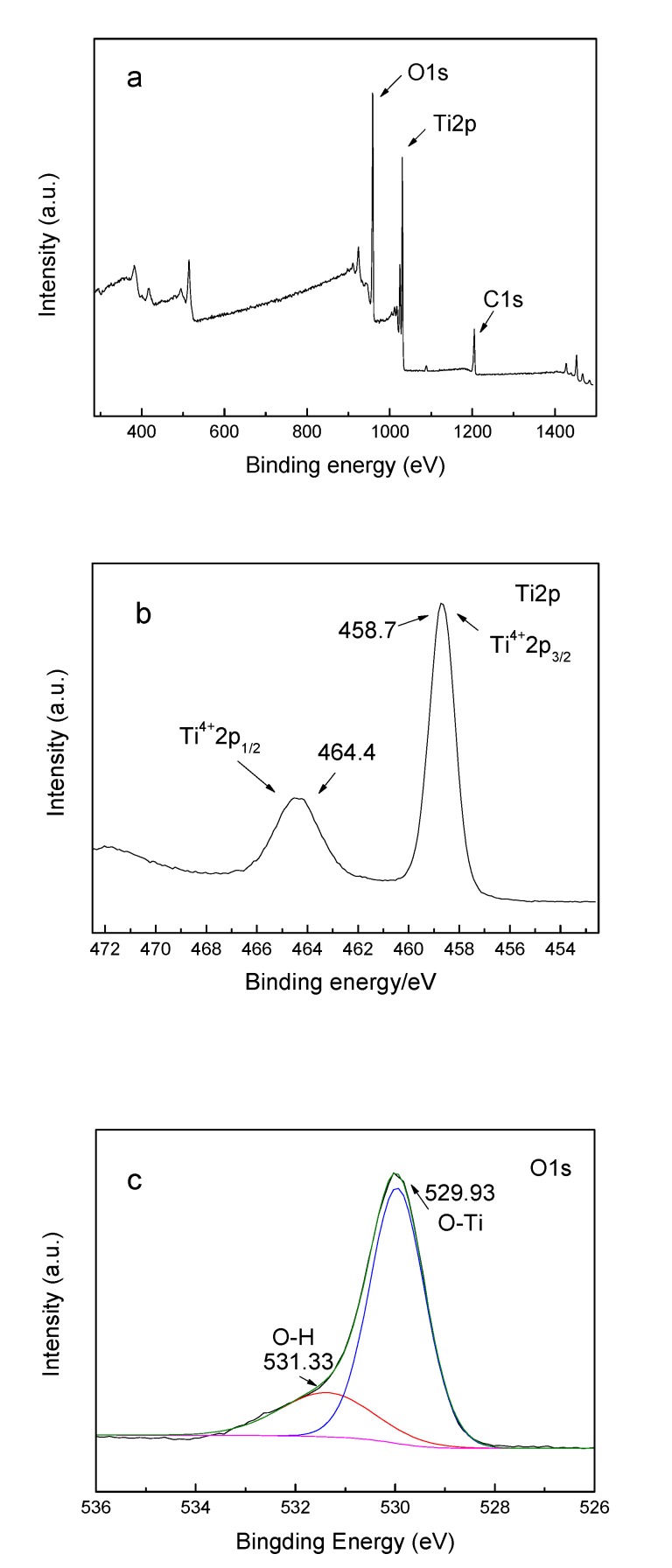
(**a**) Survey XPS spectrum of HTNTs and high-resolution spectrum for (**b**) Ti 2p and (**c**) O 1s.

By analyzing the XPS spectra of HTNTs, the chemical state of each element in the tubular catalyst was obtained. Figure 4a is a full spectrum of HTNTs, in which the peaks of Ti2p, O1s and C1s appeared in the diagram. The carbon resulted from the residual organic precursors and the XPS itself. The binding energy of Ti 2p_1/2_ and Ti 2p_3/2_ were 464.4 eV and 458.7 eV, indicating the existence of Ti^4+^. Figure 4c shows the high resolution XPS spectrum of O1s with the relative atom ratio of 55.76%. The peak of the binding energy at the position of 529.93 eV could be assigned to the crystal lattice oxygen connected by the Ti-O bond, appeared with the proportion of 77.30%. The peak of the binding energy at the position of 531.33 eV was assigned to the surface hydroxyl oxygen, with the proportion of 22.70%.

### 3.2. Electrochemical Characterization

**Figure 5:**
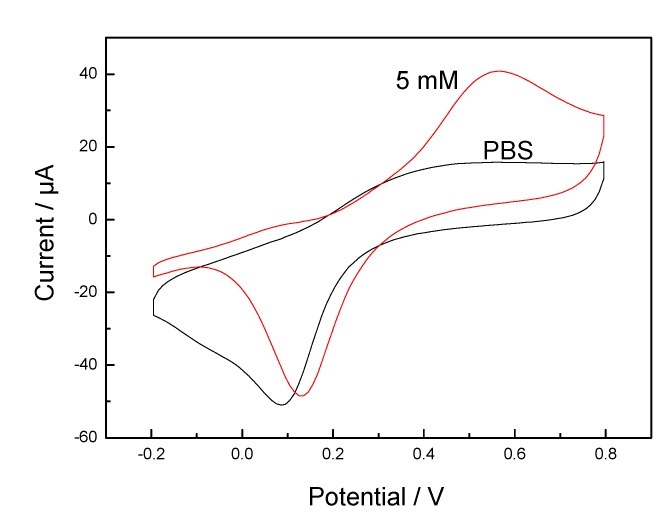
CVs recorded GOx/HTNTs/Ti electrode in PBS (0.01 M, pH = 7.4) in the absence and presence of 5 mM glucose at a scan rate of 10 mV s^−1^.

The electrochemical behavior of the GOx/HTNTs/Ti electrode is shown in Figure 5 by cyclic voltammetry. For the GOx/HTNTs/Ti electrode, cycling in PBS exhibited redox peaks at 0.1 V, which should be attributed to the reduction of Ti(IV) to Ti(III). A significant enhancement in the anodic peak current with a peak potential at about 0.55 V (vs. Ag/AgCl) can be observed after glucose was added. That should be attributed to the electro-oxidation of glucose to gluconolactone by GOx. The reaction mechanism is as follows.

GOx (ox) + glucose→gluconolactone + GOx (red)

GOx (red) → GOx (ox) + ne^−^

**Figure 6:**
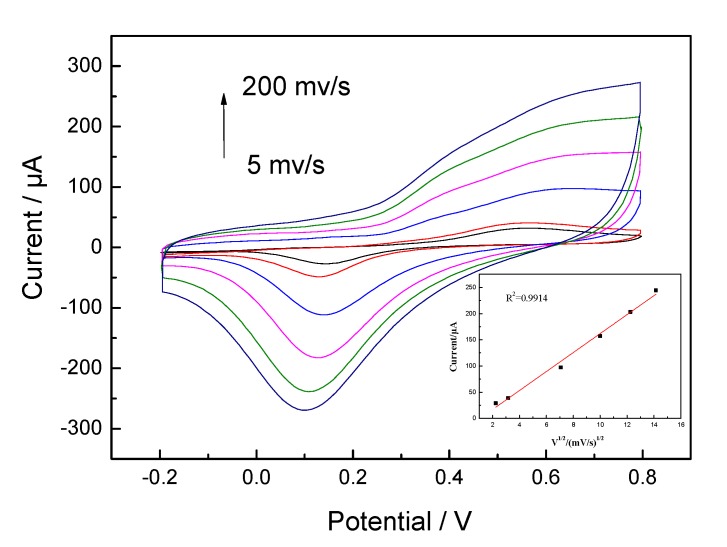
CVs recorded the GOx/HTNTs/Ti electrode at different scan rates in PBS (pH = 7.4) containing 5 mM glucose. The inserted graph showed the relationship between the peak current under the potential of +0.55 V and the square root of scan rates.

The result indicates that the GOx was immobilized on the HTNTs and the fabricated enzyme electrode revealed good detection performance for glucose. The electrochemical response of the GOx/HTNTs/Ti electrode towards glucose oxidation was investigated at different scan rates (Figure 6). The GOx/HTNTs/Ti electrode was scanned from 5 to 200 mV·s^−1^. The anodic currents were found to be proportional to the square root of the scan rate (inset of Figure 6). A good linearity between the square root of scan rate and peak current was obtained within the range of 5–200 mV·s^−1^ (correlation coefficient R^2^ = 0.9914), which indicates that the electro-oxidation of glucose was indeed diffusion-controlled when its concentration was 5 mM.

**Figure 7:**
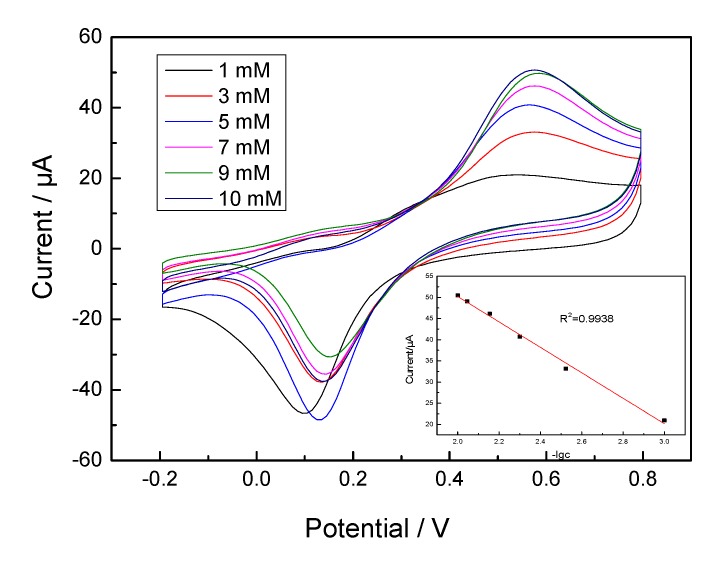
CVs of the GOx/HTNTs/Ti electrode in PBS (pH = 7.4) containing different concentrations of glucose at a scan rate of 10 mV·s^−1^. The inserted graph showed the relationship between the peak current under the potential of +0.55 V and the negative logarithm of the glucose concentration.

Typical CV curves obtained at the GOx/TNTs/Ti electrode in the glucose solution (1–10 mM) are shown in Figure 7. With the increasing of glucose concentration, the oxidation peak currents gradually increased at +0.55 V. Inserted graph shows the corresponding linear calibration curve of the GOx/HTNTs/Ti electrode. The linear range was 1–10 mM (correlation coefficient R^2^ = 0.9938). It seemed that more complex reactions occurred due to the change of glucose concentration. When the scanning rate was fixed, the current value was influenced by the diffusion ability of glucose, the reaction rate of GOx and glucose, the amount of GOx on the electrode, etc. As a result, the log relationship between the peak current and glucose concentration was formed.

**Figure 8:**
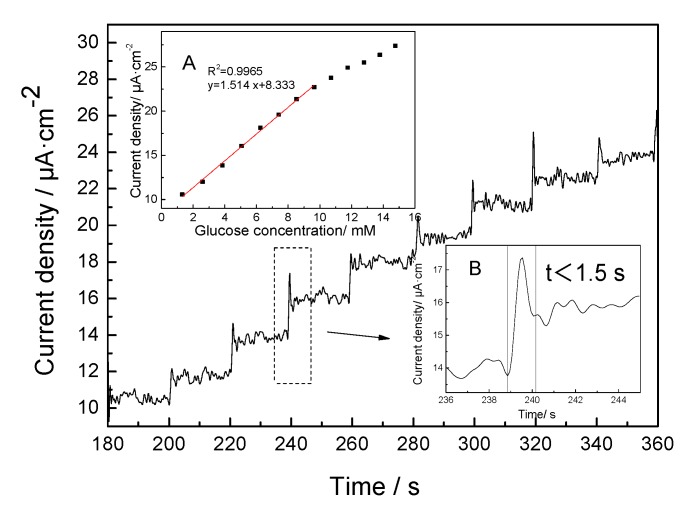
Current–time responses of the GOx/HTNTs/Ti electrode to the successive addition of glucose in PBS (pH = 7.4) under an applied potential of 0.55 V (vs. Saturated Calomel Electrode, SCE); inserted graph A showed the corresponding linear calibration curve of the GOx/HTNTs/TiO_2_ electrode as a function of glucose concentration and inserted graph B was a magnifying diagram of the dotted line square frame.

The typical amperometric responses at the GOx/HTNTs/Ti electrode for each successive addition of 0.1 M glucose are shown in Figure 8. It can be seen that the current of the electrode rose rapidly, and then stabilized rapidly after the glucose solution was added. When the concentration of glucose changed, it showed a sensitive response, and the overall current change showed a step-by-step growth. The as-prepared electrode could achieve a well-defined and step-rise current within 1.5 s (inset of Figure 8B). The results indicate that glucose was oxidized rapidly on the electrode surface. The released electrons were transferred to one end of the electron collector rapidly, and then converted into a response current. The mechanism is a glucose oxidase direct electron transfer process [31].

Table 1 summarizes the comparison of the analytical performance of various glucose biosensors. From the table, it can be seen that GOx/HTNTs/Ti sensor had a relatively wider linear range, which is a benefit for the construction of minimally invasive interventional glycemic apparatus. Compared with the response time of similar electrodes, the response time of GOx/HTNTs/Ti electrode was shorter, which should be attributed to the unique structure of the HTNTs/Ti electrode. As shown in the above SEM and TEM images, the surface of HTNTs/Ti electrode was covered by nanotubes. The 3D structure with vast pores results in more effective immobilization of GOx, more active sites for glucose and a higher reaction rate. Moreover, the HTNTs were in-situ fabricated on the surface, and the conductivity of Ti foil was good. Accompanied by the 1D structure of HTNTs, the electronic energy could be quickly transmitted to the external circuit.

The corresponding calibration curve is shown in the insert graph A of Figure 8. It can be seen that the biosensor shows a sensitive response to glucose in the concentration range of 1–10 mM. The amperometric response to glucose at 0.55 V exhibited a linear relationship with a glucose concentration in this range. Compared with the linear range reported in the literature [36], the linear range of GOx/HTNTs/Ti electrode was relatively wide. The linear regression equation is I/uA = 1.541Cg/mM + 8.333 (correlation coefficient R^2^ = 0.9965), where I is the current and Cg is the glucose concentration. The corresponding sensitivity of the glucose biosensor was 1.541 μA·mM^−^^1^·cm^−^^2^. The detection limit (LOD) of the sensor can be obtained from the formula LOD = 3 sb/S (where, sb = standard deviation of blank signal and S = sensitivity), and the calculated value was 59 μM.

However, GOx will lose its activity when pH < 2 or pH > 8, and can be irrevocably damaged at temperatures over 40 °C [37]. So, the recycling of the enzyme carrier is necessary. The self-cleaning properties of HTNTs/Ti electrode were consequently investigated. Under the illumination of a 500 Xe lamp for several seconds, the current responses of the GOx/HTNTs/Ti electrode decreased to zero. After proper irradiation, the enzyme and fouling substances on the HTNTs/Ti electrode could be entirely decomposed into CO_2_ and H_2_O to achieve self-cleaning. So, the recycling of the HTNTs/Ti electrode should be convenient.

## 4. Conclusions

In summary, the GOx/HTNTs/Ti electrode with good sensitivity performance for glucose was successfully fabricated. In this electrode, HTNTs/Ti, with a 3D structure and vast pores, was fabricated in-situ by a simple hydrothermal process, and firstly served as an enzyme carrier as well as an electron transporter to monitor glucose. The unique 3D structure resulted in effective immobilization of GOx. The in-situ fabrication and 1D structure of HTNTs could accelerate electrons transport between the biomolecules and electrode. Consequently, the response time of the GOx/HTNTs/Ti electrode was short. Moreover, the HTNTs/Ti electrode exhibited self-cleaning properties, which facilitated its recycling. Other kinds of enzymatic biosensors based on HTNTs/Ti carrier are expected.

## Figures and Tables

**Table 1:** Comparison of Various Glucose Sensors.

Configuration of Biosensor	Linear Range (Mm)	Sensitivity (μA·mM^−1^·cm^−2^)	Detection Limit (mM)	Response Time (s)	Ref.
GOx/TiO_2_-SnS_2_/Nafion/GCE	0.008–1.13 1.13–5.53	18.9	0.0018	<8	[20]
GOx/SnS_2_/Nafion	0.025–1.1	7.6	0.01	8	[32]
GOx/TiO_2_	0.005–1.32	23.2	0.002	<3	[33]
GOx/ZnO-NWs/Au/PET	0.2–2.0	19.5	<0.05	<5	[6]
Graphene/pectin-CuNPs	0.01–5.5	0.0457	0.0021	<5	[34]
GOx-AuNPs/ESM GOx/HTNTs/Ti (this work)	0.008–0.966 1–10	\ 1.541	0.0035 0.059	<3 <1.5	[35]
